# Take-Home Naloxon: Ein Baustein der Drogennotfallprophylaxe auch in Deutschland

**DOI:** 10.1007/s00103-023-03705-4

**Published:** 2023-05-26

**Authors:** Simon Fleißner, Heino Stöver, Dirk Schäffer

**Affiliations:** 1 Institut für Suchtforschung Frankfurt (ISFF), University of Applied Sciences Frankfurt, Nibelungenplatz 1, 60318 Frankfurt am Main, Deutschland; 2 Deutsche Aidshilfe e. V., Berlin, Deutschland

**Keywords:** Harm Reduction, Opioidüberdosierung, Naloxon als Nasenspray, Mortalitätsprophylaxe, Drogenbedingte Todesfälle, Harm reduction, Opioid overdose, Naloxone Nasal Spray, Mortality prophylaxis, Drug-induced death

## Abstract

Das Medikament Naloxon ist ein Opioidantagonist, der innerhalb von Minuten die (atemlähmende) Wirkung von Opioiden im Körper aufhebt. Naloxon kann dadurch zur Reduktion von Todesfällen bei Opioidüberdosierung beitragen. „Take-Home Naloxon“ (THN) ist eine Maßnahme, die vom European Monitoring Centre for Drugs and Drug Addiction (EMCDDA) und der Weltgesundheitsorganisation (WHO) empfohlen wird. Dabei werden Opioidkonsumierende und deren Angehörige, Freunde oder Bekannte im Umgang mit Naloxon geschult und für den Notfall mit dem Medikament ausgestattet.

Bisher wird THN in Deutschland überwiegend von einzelnen Einrichtungen der Suchthilfe umgesetzt. Damit das Potenzial von THN voll genutzt werden kann, ist es notwendig, die Maßnahme in Deutschland flächendeckend zu etablieren. Insbesondere in (niedrigschwelligen) Suchthilfeeinrichtungen, psychiatrischen Einrichtungen, im Rahmen von Opioidsubstitutionsbehandlungen und im Justizvollzug kann THN in das Angebot aufgenommen werden.

Dieser Diskussionsartikel greift die bisherige Entwicklung von THN in Deutschland seit 1998 auf, zeigt die Schwierigkeiten und Hindernisse einer flächendeckenden Umsetzung und stellt dar, wie THN als wirkungsvolle Public-Health-Maßnahme auch in Deutschland gelingen kann. Besonders relevant ist dies angesichts der seit 10 Jahren steigenden Zahl von Drogentodesfällen.

## Abstract (en)

Naloxone is an opioid antagonist that reverses the (respiratory-paralyzing) effects of opioids in the body within minutes. Naloxone can therefore reduce opioid overdose deaths. Take-home naloxone (THN) is an intervention recommended by the European Monitoring Centre for Drugs and Drug Addiction (EMCDDA) and the World Health Organization (WHO). It involves training opioid users and their relatives or friends in the use of naloxone and providing them with the drug in case of emergency.

So far, THN has been implemented in Germany mainly by individual addiction support facilities. In order to fully exploit the potential of THN, it is necessary to establish the measure nationwide. In particular, THN can be included in the services offered in (low-threshold) addiction support facilities, in psychiatric facilities, in the context of opioid substitution treatment, and in the correctional system.

This discussion article reviews the development of THN in Germany since 1998, highlights the difficulties and obstacles to its widespread implementation, and outlines how THN can succeed as an effective public health intervention in Germany. This is particularly relevant in view of the increasing number of drug-related deaths over the past 10 years.

## Hintergrund

Die Drogentodesfälle in Deutschland sind in den letzten 10 Jahren gestiegen. Im Jahr 2020 wurden 1581 Drogentodesfälle registriert. 572 Menschen sind am Konsum von Opioiden allein oder in Verbindung mit anderen Substanzen verstorben [1]. Auch im Jahr 2021 ist die Zahl der Drogentodesfälle weiter angestiegen auf 1826. Der Anteil der Todesfälle im Zusammenhang mit Opioiden ist weiterhin groß; allerdings lässt sich der exakte Anteil aus den aktuell veröffentlichten Zahlen nicht ermitteln [2].

Personen, die intravenös Drogen konsumieren, haben ein etwa 10fach erhöhtes Mortalitätsrisiko im Vergleich zu Personen gleichen Alters und Geschlechts aus der allgemeinen Bevölkerung [3]. 2020 war bei 36 % der Drogentodesfälle in Deutschland eine Überdosierung allein durch Opioide oder in Verbindung mit diesen todesursächlich [1]. Das European Monitoring Centre for Drugs and Drug Addiction (EMCDDA) berichtet, dass in Europa bei 76 % aller Drogentodesfälle durch Überdosierungen der Konsum von Opioiden todesursächlich war [4]. Ein besonders hohes Risiko, eine Überdosierung zu erleiden, besteht nach Abstinenzphasen, wie beispielsweise nach Haftentlassung [5] und nach Beendigung oder Abbruch einer Therapie [6, 7].

Um Todesfälle von Personen, die Opioide konsumieren, zu vermeiden, werden unterschiedliche Strategien benötigt. Das EMCDDA empfiehlt verschiedene Maßnahmen, die sich gegenseitig ergänzen. Eine dieser Maßnahmen ist „Take-Home Naloxon“ (THN; [8]). Eine Opioidüberdosierung kann zu einer (tödlichen) Atemdepression führen. Das Medikament Naloxon ist ein Opioidantagonist, der innerhalb von Minuten die Wirkung von Opioiden aufhebt. Wenn es im Falle einer Opioidüberdosierung verabreicht wird, kann dadurch das Leben der betroffenen Person gerettet werden.

Naloxon kann sich im Notfall nicht selbst verabreicht werden. Überdosierungen finden aber sehr häufig in Anwesenheit Dritter statt [9]. Diese Dritten sind meistens andere Konsumierende, enge Freunde oder Angehörige [9, 10]. Wenn diese Personen Naloxon bei sich tragen und wissen, wie es angewendet wird, können dadurch potenziell tödliche Überdosierungen verhindert werden [11, 12]. Die Opioidkonsumierenden und die Personen aus ihrem Umfeld sind größtenteils Laien, die im Rahmen von THN zunächst durch Schulungen befähigt werden müssen, eine Überdosierung zu erkennen, den Notruf abzusetzen, Erste-Hilfe-Maßnahmen durchzuführen und Naloxon zu verabreichen. Eine Naloxonschulung kann sowohl in Form einer Gruppenschulung (60–90 min) als auch als Kurzintervention (10–15 min) für Einzelpersonen durchgeführt werden. Im bayerischen Modellprojekt zum Thema Take-Home Naloxon wurde ein Schulungsprogramm evaluiert [13].

Naloxon ist in Deutschland verschreibungspflichtig und erstattungsfähig. Es ist sowohl im medizinischen als auch im nicht-medizinischen Umfeld für die Anwendung bei bekannter oder vermuteter Opioidüberdosierung zugelassen. Verschrieben werden kann das Medikament an Personen, die opioidabhängig sind. Zunächst wurde Naloxon bei der Anwendung von medizinischen Laien intramuskulär injiziert oder mit einem Zerstäuber als Off-label-Use nasal verabreicht. Seit 2018 ist in Deutschland Naloxon auch als Nasenspray (1,8 mg) verfügbar und kann zu Lasten der Krankenkassen verschrieben werden. Derzeit ist in Deutschland nur ein Präparat erhältlich. Eine Packung des Naloxon-Nasensprays enthält 2 Einheiten.

Dieser Diskussionsbeitrag zeigt auf, wie THN in Deutschland zur Reduktion der Todesfälle im Zusammenhang mit Opioiden umgesetzt werden kann. Dazu wird zunächst die Geschichte von THN dargestellt. Anschließend wird die Entwicklung von THN in Deutschland nachgezeichnet und die bisherigen Schwierigkeiten werden benannt, um darauf aufbauend aufzuzeigen, in welchen Angeboten THN-Projekte eingeführt werden sollten, damit THN in Deutschland erfolgreich sein kann. Der Beitrag schließt mit einem Ausblick, welche Schritte in Deutschland für eine Umsetzung von THN noch erforderlich sind.

## Die Entwicklung von Take-Home Naloxon

Naloxon wurde in den 1960er-Jahren in New York das erste Mal synthetisiert und in den USA patentiert. In den 1970er-Jahren wurde das Medikament in die klinische Praxis übernommen und in den 1990er-Jahren wurden die ersten Vorschläge zur Take-Home-Nutzung von Naloxon veröffentlicht [14–16]. Die ersten THN-Pilotprojekte wurden 1998 unter anderem in Berlin und New York durchgeführt. Dabei wurden in Berlin innerhalb von 16 Monaten 124 Opioidkonsumierende geschult und mit Naloxon ausgestattet [17]. Nach diesen ersten Pilotprojekten wurden THN-Programme auch in deutlich größerem Umfang realisiert und die Wirksamkeit untersucht.^1^ Eines der ersten staatlichen THN-Programme wurde in Schottland ins Leben gerufen. Zwischen April 2011 und März 2014 wurden dabei 11.270 Naloxonkits^2^ ausgegeben. Bei einem Vergleich der Zeiträume 2006–2010 und 2011–2014 wurde eine Reduktion der Todesfälle im Zusammenhang mit Opioiden festgestellt, die unter anderem auch auf das staatliche THN-Programm zurückzuführen war [18].

Eine randomisierte kontrollierte Studie zu THN konnte aus forschungspraktischen und ethischen Gründen bisher nicht realisiert werden, jedoch können die Bradford-Hill-Kriterien [19] bei der Beurteilung von Public-Health-Maßnahmen als Leitlinie dienen. Voneinander unabhängig kommen 2 systematische Übersichtsarbeiten zu dem Ergebnis, dass nach den Bradford-Hill-Kriterien von einem kausalen Zusammenhang zwischen THN und der Mortalität von Opioidkonsumierenden auszugehen ist [11, 12]. Beide Übersichtsarbeiten empfehlen die Ausweitung von THN-Programmen und gehen davon aus, dass dadurch die Mortalität von Menschen, die Opioide konsumieren, wirkungsvoll gesenkt werden kann [11, 12].

## Take-Home-Naloxon-Projekte in Deutschland

Eine vollständige Übersicht von THN-Projekten in Deutschland existiert nicht, weshalb im Folgenden vermutlich nicht alle THN-Projekte in Deutschland genannt werden können. Nachdem der Verein „Fixpunkt e. V.“ 1998 in Berlin als Erster THN umgesetzt hat [17], wurden in Deutschland nur vereinzelt und regional stark begrenzt weitere Projekte ins Leben gerufen. Das Interesse an Drogennotfalltrainings und Naloxonvergabe war in Deutschland eher gering [20]. 2013 hat in Frankfurt die erste nationale Konferenz zum Thema THN stattgefunden und 2017 folgte eine weitere in München.

Von 2014 bis 2016 hat die Integrative Drogenhilfe in Frankfurt Naloxonschulungen mit anschließender Naloxonverschreibung angeboten. Nachdem 2016 die ärztliche Kooperation weggefallen ist, wurde das Angebot ausgesetzt [21]. In Köln wurden 2016 durch den Verein für innovative Drogenselbsthilfe Vision e. V. und JES NRW (Selbsthilfe von Junkies, Ehemaligen und Substituierten) in verschiedenen Städten Nordrhein-Westfalens Schulungen mit Verschreibung von Naloxon angeboten. Damit wurden 106 Personen erreicht [22]. Im gleichen Jahr starteten die ersten Naloxonschulungen in München, durchgeführt von Condrobs e. V. Hierfür mussten in 2 Jahren Vorbereitungszeit zuerst noch Mitarbeitende und Ärzt:innen von Naloxon als Take-Home überzeugt werden [23]. 2017 hat die Drogenhilfe Saarbrücken ein THN-Projekt, das durch das saarländische Ministerium für Soziales, Gesundheit, Frauen und Familie gefördert wurde, ins Leben gerufen [24]. Eine Übersicht von 2018 zeigt, dass in Münster, Köln, Mannheim, Saarbrücken, Tübingen, München und Berlin bereits THN-Schulungen angeboten wurden. In Düsseldorf, Frankfurt, Nürnberg, Regensburg, Ulm, Ingolstadt und Augsburg wurde die Umsetzung zu diesem Zeitpunkt geplant [25].

Im Jahr 2018 wurde das bayerische Naloxon-Modellprojekt begonnen. Das Ziel war es, zu prüfen „… unter welchen Voraussetzungen ein THN-Programm in Bayern medizinisch sicher, effektiv und rechtssicher als fester Bestandteil der Drogenhilfe und als Möglichkeit zur Verhinderung von akuten Drogentodesfällen implementiert werden kann“ [13]. Damit wurde ein zwar auf Bayern begrenztes, aber einrichtungsübergreifendes THN-Programm mit öffentlichen Mitteln durchgeführt. Das Naloxon wurde in diesem Projekt über Privatrezepte verschrieben. Teilgenommen haben niedrigschwellige Suchthilfeeinrichtungen und einige Justizvollzugsanstalten. Es wurden ca. 500 Personen im Rahmen des Projektes erreicht [13].

Im Jahr 2021 wurde mit dem aktuell laufenden Bundesmodellprojekt NALtrain (Konzeption, Umsetzung und Evaluation eines Modellprojekts zur Durchführung deutschlandweiter qualitätsgesicherter Take-Home-Naloxon-Schulungen)^3^ ein durch das Bundesministerium für Gesundheit gefördertes THN-Programm umgesetzt. NALtrain ist ein 3‑jähriges Praxisprojekt (07/2021 bis 06/2024), das THN als Regelangebot in der deutschen Suchthilfe etablieren möchte. Dazu werden 800 Mitarbeitende aus 400 Drogen- und Suchthilfeeinrichtungen ausgebildet, um für Opioidkonsumierende und Menschen in Substitutionsbehandlung Naloxonschulungen anzubieten. Außerdem werden Kontakte und Kooperationen mit Ärzt:innen unterstützt. Bis 06/2024 sollen auf diese Weise 10.000 Opioidkonsumierende oder Menschen in Substitutionsbehandlung mit Naloxonschulungen erreicht werden und Naloxon erhalten. Da Angehörigen, Bekannten und Freunden in Deutschland bisher kein Naloxon verschrieben werden kann, werden diese nicht explizit durch das Projekt adressiert.

Im Rahmen von NALtrain ist keine finanzielle Unterstützung der umsetzenden Einrichtungen vorgesehen. Bisher wurde lediglich in Bayern eine allgemeine Finanzierung für Naloxonschulungen geschaffen. In Baden-Württemberg und in Nordrhein-Westfalen wurde die Finanzierung von THN im Justizvollzug vonseiten der Justizministerien bewilligt. Das Naloxon kann bei indikationsgemäßer Verschreibung durch die Krankenkassen finanziert werden. Im Projekt NALtrain wird darauf hingewirkt, dass neben Bayern weitere Bundesländer eine Finanzierung von THN beschließen und sich das Projekt so verstetigen kann. Ohne eine solche Finanzierung könnte es für viele Einrichtungen herausfordernd werden, den zusätzlichen personellen Aufwand aufrechtzuerhalten und auch Nichtversicherten zu ermöglichen, in den Besitz von Naloxon zu kommen.

Damit THN in Deutschland erfolgreich sein kann, ist es notwendig, die Mitarbeitenden aus allen Einrichtungen der Suchthilfe zu qualifizieren, die Opioidkonsumierenden und Menschen in Substitutionsbehandlung als potenzielle Anwender:innen von Naloxon zu schulen und die Verschreibung von Naloxon im Anschluss an die Schulungen zu gewährleisten (Infobox 1).

## Settings und Zielgruppen für Take-Home Naloxon in Deutschland

Bereits 1996 haben Strang et al. mögliche Zielgruppen für THN aufgelistet [15]. Damit möglichst viele der Zielgruppen erreicht werden, reicht es nicht aus, THN-Schulungen in der niedrigschwelligen Drogenhilfe anzubieten. Es bieten sich verschiedene Settings an, welche im Folgenden mit ihren jeweiligen Möglichkeiten zur erfolgreichen Umsetzung von THN in Deutschland dargestellt werden (Tab. 1).

**Table 1:** 

Setting	Chancen	Schwierigkeiten
1. Niedrigschwellige THN-Angebote in der Drogenhilfe	Erreicht Personen mit einem vergleichsweise hohen Risiko, eine Überdosierung zu erleiden	Schulungstermine werden häufig nicht eingehalten
	Verbreitung in der örtlichen Szene	Verschreibung von Naloxon nur durch Ärzt:innen möglich
		Naloxon muss in der Apotheke vorrätig sein
		Klient:innen teilweise nicht krankenversichert
		Nicht alle Klient:innen interessiert
2. THN im Rahmen einer Opioidsubstitutionsbehandlung	Ärztliche Anbindung bereits vorhanden	Ablehnung der Patient:innen aus Angst vor Konsequenzen in der Substitutionstherapie
	Übernahme der Kosten für das Naloxon-Nasenspray durch die Krankenkassen	Ablehnung durch Patient:innen, da scheinbar im Widerspruch zum Behandlungsziel
	Schulungstermine in der Praxis einfacher durchzuführen	
3. THN in psychiatrischen Einrichtungen, Entzugs- und Entwöhnungskliniken sowie im Maßregelvollzug	Sehr gute Erreichbarkeit der Zielgruppe (z. B. Naloxonschulung als „normales“ Angebot der Behandlung)	Ablehnung durch Patient:innen, da scheinbar im Widerspruch zum Therapieziel
	Gute Erreichbarkeit einer Zielgruppe mit erhöhtem Überdosierungsrisiko	
	Ärztliche Anbindung	
	Einfache Verschreibung von Naloxon und einfacher Zugang zum Medikament	
4. THN im Justizvollzug	Sehr gute Erreichbarkeit der Zielgruppe	Evtl. kein Interesse der Zielgruppe
	Gute Erreichbarkeit einer Zielgruppe mit erhöhtem Überdosierungsrisiko	Bereitschaft der Justizministerien fraglich

*THN* Take-Home Naloxon

### 1. Niedrigschwellige THN-Angebote in der Drogenhilfe

Bisher werden THN-Schulungen vor allem im niedrigschwelligen Bereich wie in Kontaktläden und Drogenkonsumräumen angeboten. Gerade hier können aktuell Konsumierende erreicht werden. Es besteht die große Chance, Naloxon in der örtlichen „Szene“ zu verbreiten und damit den Opioidkonsumierenden bei einer Überdosierung – sowohl im öffentlichen als auch im privaten Raum – zu helfen. Im niedrigschwelligen Bereich gibt es jedoch 2 besondere Schwierigkeiten bei der Umsetzung der Maßnahme.

Die erste Hürde ist die Erreichbarkeit der Klient:innen. Gerade feste Schulungstermine werden von ihnen häufig, trotz Anmeldung, nicht wahrgenommen. Ein flexibleres Angebot durch kurze Einzelschulungen (10–15 min), sogenannte Kurzinterventionen, erscheint hier sinnvoll [26], fordert aber auch von den Mitarbeitenden eine größere Flexibilität.

Die zweite Schwierigkeit besteht aufgrund der Verschreibungspflicht von Naloxon. Bei einer spontanen Einzelschulung muss für die Verschreibung zusätzlich ein Arzt oder eine Ärztin anwesend sein und die Apotheke muss das Naloxon-Nasenspray vorrätig haben. Der kurzfristige Kontakt zu Ärzt:innen ist aber häufig nicht möglich. Lösungsmöglichkeiten sind eine enge Kooperation mit substituierenden Ärzt:innen oder die Durchführung der Schulungen direkt in der ärztlichen Praxis, um so eine unmittelbare Verschreibung zu ermöglichen.

Ein weiteres Problem ist, dass das Interesse der Klient:innen an einer Schulung nicht immer gegeben ist. Klient:innen sehen beispielsweise für sich selbst keinen Bedarf, an einer Naloxonschulung teilzunehmen. Deshalb kann ein häufiges Werben für Naloxon hilfreich sein, beispielsweise mit der Frage: „Hast du 15 min Zeit, um zukünftig deinem Freund bei einer Überdosierung helfen zu können?“ Bezüglich der Erreichbarkeit kommt erschwerend hinzu, dass nicht alle Klient:innen krankenversichert sind, weshalb die Finanzierung von Naloxon durch die Krankenkassen nicht immer gegeben ist.

### 2. THN im Rahmen einer Opioidsubstitutionsbehandlung

Durch eine Opioidsubstitutionsbehandlung sinkt die Mortalität der Betroffenen deutlich. Trotzdem versterben auch Personen in Substitutionsbehandlung an einer Überdosierung (z. B. durch Beikonsum). Insbesondere in den ersten 2–4 Wochen der Behandlung und kurz nach dem Ausscheiden aus der Substitutionsbehandlung besteht ein erhöhtes Mortalitätsrisiko [27–29]. Da durch die Behandlung eine ärztliche Anbindung bereits gegeben ist, bieten sich eine Naloxonschulung und die anschließende Verschreibung des Medikaments hier besonders an. Die Indikation für die Verschreibung des Naloxon-Nasensprays beinhaltet auch Personen in Substitutionsbehandlung, weshalb die Kosten für das Nasenspray durch die Krankenkassen getragen werden.

Die Etablierung der Naloxonschulungen und der Verschreibung im Rahmen von Opioidsubstitutionsbehandlungen könnte einen entscheidenden Beitrag für die erfolgreiche Umsetzung von THN in Deutschland leisten, da hier besonders viele Menschen erreicht werden können. Insbesondere direkt zu Beginn einer Behandlung erscheint die Verschreibung von Naloxon zielführend. Es ist allerdings auch damit zu rechnen, dass einige Patient:innen die Naloxonschulung ablehnen. Zum einen könnten Konsequenzen für die eigene Behandlung befürchtet werden, da der Bedarf an Naloxon auf vermeintlichen Beikonsum hinweisen könnte, und zum anderen erscheint THN im Widerspruch zum Behandlungsziel der Patient:innen zu stehen. Hier kann Aufklärung durch die behandelnden Ärzt:innen Vorbehalte abbauen. Die Schulung der Patient:innen kann im Rahmen der psychosozialen Betreuung als Kurzintervention stattfinden oder auch als Gruppenangebot beworben werden. Auch die Kooperation mit externen Suchthilfeeinrichtungen ist denkbar und wird in einigen Praxen bereits erfolgreich durchgeführt.

### 3. THN in psychiatrischen Einrichtungen, Entzugs- und Entwöhnungskliniken sowie im Maßregelvollzug

Nach Entlassung aus einer Klinik, egal ob nach regulär beendeter oder abgebrochener Behandlung, ist das Risiko einer Überdosierung besonders hoch [6]. Patient:innen vor der Entlassung zu schulen und mit Naloxon auszustatten würde also eine sehr vulnerable Zielgruppe erreichen. Gleichzeitig könnte die Therapie ein Setting bieten, in dem zum einen die Themen „Überdosierung“ und „Drogennotfall“ besprochen werden können und zum anderen der ärztliche Kontakt und die Versorgung mit Naloxon sehr einfach möglich sind.

THN kann aber auch hier den Patient:innen und Behandler:innen als Widerspruch zum Therapieziel erscheinen [30]. Es gilt also, die Themen „Überdosierung“ und „Drogennotfall“ passend in die Therapie einzubeziehen. Dem erhöhten Risiko einer Überdosierung nach Beendigung oder Abbruch kann damit begegnet werden. Denkbar ist es, das Thema in eine Gruppenschulung zur „Rückfallprophylaxe“ aufzunehmen. Auch bei abstinenzorientierten Therapien kann Naloxon zum Thema gemacht werden. Es kann als freiwilliges Angebot integriert, aber auch als fester Bestandteil der Behandlung angeboten werden. Die Mitgabe von Naloxon bei Beendigung oder Abbruch der Therapie sollte ermöglicht werden. Denkbar ist auch eine Versorgung mit Naloxon direkt im Anschluss an eine Naloxonschulung noch während der Therapie. Diese Vorschläge richten sich ebenfalls an Einrichtungen des Maßregelvollzuges nach § 64 StGB.

### 4. THN im Justizvollzug

Auch in den ersten 48 h nach Haftentlassung ist das Risiko einer Überdosierung besonders hoch [31]. Die „Bundeseinheitliche Erhebung zur stoffgebundenen Suchtmittelproblematik im Justizvollzug“ ergab, dass 44 % der Inhaftierten Substanzabhängigkeit oder Substanzmissbrauch aufweisen. Davon sind bei 13 % der männlichen Inhaftierten und bei 28 % der weiblichen Inhaftierten Opioide die Hauptsubstanz [32]. Bird et al. kommen zu dem Ergebnis, dass das nationale THN-Programm für Menschen in Haft in Schottland die Drogentodesfälle in Zusammenhang mit Opioiden in den ersten 4 Wochen nach Haftentlassung um 36 % reduziert hat [33].

Gerade der Justizvollzug bietet einen geeigneten Rahmen für Naloxonschulungen und die Mitgabe von Naloxon bei Haftentlassung. Es können Gruppenschulungen angeboten werden, bei denen Inhaftierte gut als Peers eingebunden werden könnten. Horsburgh und McAuley berichten von der Umsetzung in Schottland, dass Gruppenschulungen für die dortigen Gefängnisse einen zusätzlichen Aufwand bedeuten, weshalb sich hier eher Kurzinterventionen für Einzelne anbieten würden [34]. Das bayerische Modellprojekt hingegen berichtet, dass Gruppenschulungen gut geeignet waren und die Motivation der Gefangenen hoch war [13]. Die Naloxonschulungen können sowohl durch Bedienstete der Haftanstalt selbst (z. B. medizinischer Dienst, sozialer Dienst) angeboten werden oder aber auch in Kooperation mit externen Suchthilfeeinrichtungen. Die Zusammenarbeit mit der medizinischen Abteilung ist in jedem Fall für die Ansprache der Betroffenen und die Beschaffung des Naloxons wichtig.

Nicht alle Inhaftierten zeigen Interesse an THN. Eine mehrmalige Ansprache sowohl zu Beginn der Haftstrafe als auch 6–12 Wochen vor Entlassung kann zu einer größeren Beteiligung beitragen. Bei Gefangenen mit ungewissem Entlassungstermin (Untersuchungshaft) sollte die Naloxonschulung möglichst direkt zu Beginn stattfinden. Die Ausgabe von Naloxon sollte bei Haftentlassung zusammen mit der Habe erfolgen. Sie ist ein entscheidender Bestandteil, da nur so sichergestellt werden kann, dass die betroffene Person in den Tagen nach Haftentlassung Naloxon zur Verfügung hat.

In Bayern, Baden-Württemberg und Nordrhein-Westfalen werden in einigen Justizvollzugsanstalten bereits Schulungen angeboten. Für die Umsetzung von THN in Haft ist die Bereitschaft der Justizministerien und Justizvollzugsanstalten notwendig. THN-Projekte in Haft können einen entscheidenden Beitrag zur Verhinderung von Drogentodesfällen in Deutschland leisten. Das bayerische Modellprojekt hat gezeigt, dass eine Umsetzung von THN im Justizvollzug möglich ist [13].

### Die aktuelle Umsetzung und weitere mögliche Zielgruppen

Die 4 genannten Ansatzpunkte könnten sich besonders gut eignen, um THN-Projekte umzusetzen und Personen, die Opioide konsumieren, zu erreichen. In Deutschland ist THN bisher in der niedrigschwelligen Suchthilfe am weitesten verbreitet. Einige Justizvollzugsanstalten und einige substituierende Ärzt:innen haben ebenfalls begonnen, THN in ihre Arbeit zu integrieren. Auch psychiatrische Einrichtungen beteiligen sich am Bundesmodellprojekt NALtrain. Das heißt, es gibt in den verschiedenen Bereichen bereits vielversprechende Anfänge. Damit THN seine Wirkung voll entfalten kann, sollte die Umsetzung nicht auf einzelne Einrichtungen oder Bereiche der Suchthilfe beschränkt bleiben. Es sollten möglichst vielfältige Zugänge genutzt und THN strukturell verankert werden.

Außerdem bilden Angehörige, Bekannte und Freunde von Opioidkonsument:innen eine weitere Zielgruppe, die in THN-Programme eingebunden werden könnte. Bei dieser Personengruppe besteht eine erhöhte Wahrscheinlichkeit, bei einer Überdosierung anwesend zu sein [6]. Sie zu schulen und mit Naloxon auszustatten ist also ebenfalls zielführend. Beispielsweise könnten in psychiatrischen Einrichtungen Angehörige angesprochen und eingebunden werden, aber auch in Kontaktläden oder Beratungsstellen. In Deutschland ist es bisher jedoch rechtlich nicht möglich, Naloxon auch an Angehörige, Bekannte und Freunde zu verschreiben. Auch sind die Angebote der Sucht- und Drogenhilfe kaum auf diese Zielgruppe ausgerichtet. Ihre Erreichbarkeit mit Naloxonschulungen ist dadurch erschwert.

## Voraussetzungen für eine flächendeckende Umsetzung in Deutschland

Damit THN in Deutschland flächendeckend eingeführt werden kann, müssen alle Bereiche der Suchthilfe eingebunden werden. Dazu ist eine Qualifizierung von Mitarbeitenden aus allen Bereichen erforderlich, damit diese wiederum selbst Schulungen anbieten können. Das können Sozialarbeitende, Personal aus dem Justizvollzug (z. B. Sozialdienst oder medizinischer Dienst) oder auch medizinische Fachangestellte sein. Darüber hinaus sollte THN im Bereich der Suchtmedizin zu einer standardisierten Behandlung bei Menschen mit Opioidkonsum werden und nicht die Ausnahme sein. Selbstverständlich ersetzt die Naloxongabe bei Überdosierung nicht den Notruf oder eine andere medizinische Behandlung. Es zeigt sich aber, dass bei einem Drogennotfall nicht immer der Notruf abgesetzt wird [13, 35] und damit Naloxon mitunter die einzige Möglichkeit ist, das Leben der betroffenen Person zu retten. Auch das Risiko, Beikonsum oder risikoreicheren Konsum zu fördern, ist einem systematischen Review von Tse et al. zufolge nicht haltbar [36].

Bei der Umsetzung von THN ist die Bereitschaft von Mediziner:innen erforderlich, an dieser Public-Health-Maßnahme mitzuwirken, da nur sie die benötigten Rezepte ausstellen dürfen. Gerade die Notwendigkeit einer ärztlichen Kooperation stellt eine große Hürde für die flächendeckende Umsetzung von THN dar. Alternativ könnte die Aufhebung der Verschreibungspflicht die Umsetzung vereinfachen. In Italien beispielsweise ist Naloxon nicht verschreibungspflichtig [37, 38] und im Vereinigten Königreich können auch Suchthilfeeinrichtungen Naloxon an alle Personen ausgeben, bei denen eine erhöhte Wahrscheinlichkeit besteht, Zeuge einer Überdosierung zu werden [38].

Für eine flächendeckende Versorgung in Deutschland könnte die Aufhebung der Verschreibungs- und Apothekenpflicht bei gleichzeitiger Finanzierung durch die Länder zielführend sein. Nach einer Naloxonschulung könnte dann direkt Naloxon ausgegeben werden. Dies erscheint bisher aber nicht realistisch. Naheliegender erscheint es, Naloxon zwar von der Verschreibungspflicht auszunehmen, aber es dennoch weiterhin erstattungsfähig zu belassen, was auch eine der Empfehlungen im Endbericht des bayerischen Modellprojekts war [13]. Bislang wird Naloxon in Deutschland nach wie vor häufig nur auf Privatrezept verschrieben, obwohl es auch zu Lasten der Krankenkassen an Personen mit Opioidabhängigkeit verschrieben werden kann. Beim Privatrezept wird die Finanzierung des Medikaments meist von den Einrichtungen der Suchthilfe übernommen. Zusätzlich fehlt es häufig an einer Finanzierung der Organisation und praktischen Durchführung von THN. Bisher gibt es nur in Bayern eine finanzielle Förderung. Damit und mit dem Engagement der dortigen Einrichtungen konnte das bayerische Modellprojekt erfolgreich durchgeführt und THN bereits in vielen bayerischen Einrichtungen etabliert werden. Wichtige Erfordernisse für eine flächendeckende Einführung von THN in Deutschland sind in Infobox 2 dargestellt.

## Fazit

THN könnte auch in Deutschland zu einer Reduktion der Mortalität von opioidkonsumierenden Personen und Personen in Substitutionstherapie führen. Durch Naloxon als Take-Home in Verbindung mit einer entsprechenden Schulung können potenziell tödlich verlaufende Opioidüberdosierungen durch anwesende Dritte behandelt und Leben gerettet werden. Bisher gibt es in Deutschland noch keine flächendeckende Versorgung mit THN. Mit Einführung von THN in der Suchtkrankenhilfe, der Substitutionsbehandlung, den psychiatrischen Einrichtungen, den Entzugs- und Entwöhnungskliniken und dem Justizvollzug als Standard könnte die Zielgruppe gut erreicht werden. THN als Maßnahme der öffentlichen Gesundheitsfürsorge umzusetzen wird sowohl von der WHO als auch von der EMCDDA empfohlen. Auch aufgrund der steigenden Drogentodeszahlen der letzten Jahre in Deutschland sollten alle empfohlenen Maßnahmen zur Reduktion der Mortalität von drogengebrauchenden Menschen in Deutschland ausgeschöpft werden. THN ist eine Maßnahme, welche noch deutlich ausgebaut werden kann und nach wissenschaftlichen Erkenntnissen Leben rettet [11, 12].

### Infobox 1 Für die Umsetzung von „Take-Home Naloxon“ (THN) werden benötigt

- Qualifikation von Mitarbeitenden der Suchthilfe
- Schulung von Opioidkonsumierenden und Menschen in Substitutionsbehandlung als potenzielle Anwender:innen von Naloxon
- Die Verschreibung von Naloxon und die Versorgung damit

### Infobox 2 Wichtige Erfordernisse für eine flächendeckende Einführung von „Take-Home Naloxon“ (THN) in Deutschland

- Einbindungen aller Bereiche der Suchthilfe
- Finanzielle Förderung von THN
- Erleichterte Abgabe von Naloxon (z. B. durch zertifizierte Drogenhilfeeinrichtungen)
- Erweiterung des Indikationsbereichs (für Angehörige, Sozialarbeiter etc.)

## Footnotes

1 Für einen ausführlicheren zeitlichen Ablauf von THN-Programmen siehe [9].

2 Ein Naloxonkit wird meist von der Suchthilfeeinrichtung ausgegeben und enthält in einer Metalldose beispielsweise: Naloxon (als Nasenspray oder als Spritzen), Beatmungstuch, Anleitung, Einmalhandschuhe.

3 Weitere Informationen finden Sie online: https://www.naloxontraining.de/naltrain/.
